# Inactivation of the MAPK signaling pathway by *Listeria monocytogenes* infection promotes trophoblast giant cell death

**DOI:** 10.3389/fmicb.2015.01145

**Published:** 2015-10-16

**Authors:** Masanori Hashino, Masato Tachibana, Takashi Nishida, Hideki Hara, Kohsuke Tsuchiya, Masao Mitsuyama, Kenta Watanabe, Takashi Shimizu, Masahisa Watarai

**Affiliations:** ^1^The United Graduate School of Veterinary Science, Yamaguchi University, Yoshida CampusYamaguchi, Japan; ^2^Division of Biomedical Food Research, National Institute of Health SciencesTokyo, Japan; ^3^Department of Microbiology, Graduate School of Medicine, Kyoto UniversityKyoto, Japan; ^4^Department of Pathology and Comprehensive Cancer Center, University of Michigan Medical SchoolAnn Arbor, MI, USA; ^5^Division of Immunology and Molecular Biology, Cancer Research Institute, Kanazawa UniversityKanazawa, Japan; ^6^Graduate School of Advanced Integrated Studies in Human Survivability, Kyoto UniversityKyoto, Japan; ^7^Laboratory of Veterinary Public Health, Joint Faculty of Veterinary Medicine, Yamaguchi University at YamaguchiYamaguchi, Japan

**Keywords:** *Listeria monocytogenes*, MAPK, infection, abortion, placenta, trophoblast

## Abstract

*Listeria monocytogenes* has a well-characterized ability to cross the placental barrier, resulting in spontaneous abortion and fetal infections. However, the mechanisms resulting in infection-associated abortion are not fully understood. In this study, we demonstrate that the dephosphorylation of MAPK family proteins caused by *L. monocytogenes* infection of trophoblast giant (TG) cells, which are placental immune cells, contributes to infectious abortion. Dephosphorylation of c-Jun, p38, and ERK1/2 was observed in infected TG cells, causing the downregulation of cytoprotective heme oxygenase (HO)-1. Blocking the dephosphorylation of proteins, including MAPK family proteins, inhibited the decrease in HO-1 expression. Treatment with MAPK inhibitors inhibited bacterial internalization into TG cells. Moreover, Toll-like receptor 2 involved in the expression of MAPK family proteins. Infection with a listeriolysin O-deleted mutant impaired dephosphorylation of MAPK family proteins in TG cells and did not induce infectious abortion in a mouse model. These results suggest that inactivation of the MAPK pathway by *L. monocytogenes* induces TG cell death and causes infectious abortion.

## Introduction

Human listeriosis is a food-borne disease resulting from the ingestion of contaminated food, such as dairy products, vegetables, raw seafood, poultry, and processed meat (Hernandez-Milian and Payeras-Cifre, 2014). *Listeria monocytogenes* is the Gram-positive bacterium and causative agent of listeriosis. In humans, listeriosis causes gastroenteritis, maternofetal infections, and meningoencephalitis due to *L. monocytogenes* ability to cross the blood–brain, placental, and intestinal barriers. An important virulence feature of *L. monocytogenes* is its ability to escape from the killing mechanisms of phagocytic host cells, such as macrophages (Stavru et al., 2011a; Ribet and Cossart, 2015).

Pregnancy causes a widely suppression of the adaptive immune system, characterized by a decreased cell-mediated immunity and the suppressed response of cytotoxic T cells (Gluhovschi et al., 2015). Maternal rejection of the fetus is prevented by the immunosuppressed state; however, it has the unexpected effect of increasing maternal susceptibility to abortion-inducing pathogens (Dhama et al., 2015). *L. monocytogenes* is an intracellular pathogen and its immunity is principally mediated by cellular immune responses (Parmer, 2004). In our previous study, the abortion was observed in pregnant mouse model infected with abortion-inducing bacteria, such as *L. monocytogenes* and *Brucella abortus* (Kim et al., 2005; Tachibana et al., 2008, 2011). In *Brucella* infection, compared to other organs, a large amount of bacterial colonization was found in the placenta, especially in the placental trophoblast giant (TG) cells. In contrast, an intracellular replication-defective mutant failed to induce abortion in a pregnant mouse model (Kim et al., 2005). In addition, infection of *B. abortus* induced a transient increase in interferon-γ (IFN-γ) levels in pregnant mice. Furthermore, this transient IFN-γ production leads to infectious abortion, and depletion of IFN-γ by neutralization inhibits infectious abortion (Kim et al., 2005). These reports of *B. abortus* infection imply that bacterial internalization and intracellular replication in TG cells are both key aspects in abortion and that TG cells are closely associated with the evasion of maternal immune rejection.

Trophoblast giant cells are essential for the establishment of pregnancy. TG cells are polyploid cells differentiated from trophoblast stem (TS) cells. TG cells have diverse functions that are crucial for implantation and subsequent placental function such as vasculature remodeling and uterine immune system (Hu and Cross, 2010). They form the fetal component of the placenta (Parast et al., 2001). In particular, TG cells regulate maternal spiral artery remodeling and maternal blood flow into the placenta in mice (Hu and Cross, 2010). TG cells in the mouse placenta are analogous to extravillous cytotrophoblast (EVT) cells in the human placenta (Baczyk et al., 2004). *L. monocytogenes* and *Toxoplasma gondii* enter into EVT cells preferentially in primary human placental organ cultures (Robbins et al., 2010, 2012). Trophoblast cells show phagocytic feature. Trophoblast cells phagocytose stroma and uterine epithelial cells and invade maternal tissue during implantation (Welsh and Enders, 1987). Several of the mechanisms contributed to phagocytic ability of trophoblast cells have been published (Drake and Rodger, 1987), however, the detail process is still unclear. Another report showed that trophoblast cells have the ability of bacterial uptake and that IFN-γ treatment enhaces this activity (Amarante-Paffaro et al., 2004). Therefore, trophoblast cells may have phagocytic activity against pathogenic agents in a same way of macrophages. These studies suggest that trophoblast cells play roles in the placental defense system as well as in the development and maintenance of placenta.

Various cell types, such as dendritic cells (Guzmán et al., 1996), lymphocytes (Merrick et al., 1997), and hepatocytes (Rogers et al., 1996), are induced cell death *in vitro* and *in vivo* by *L. monocytogenes* infection. The pore-forming toxin listeriolysin O (LLO) play important role in cell death induced by *L. monocytogenes*. LLO is the key factor responsible for degradation of the vacuole and escaping into the cytosol. In previous studies, we demonstrated that *Listeria* and *Brucella* infections are associated with the death of TG cells. We also found that reduction of heme oxygenase (HO)-1 expression by bacterial infection enhanced infectious abortions *in vivo* and cell death *in vitro* (Tachibana et al., 2008, 2011). HO-1 plays key roles in cytoprotection, antioxidation, and anti-inflammation. The majority of HO-1’s physiological functions are associated with its enzymatic activity in heme catabolism (Hegazi et al., 2005; Nakahira et al., 2006). HO-1 deficiency causes an increased pro-inflammatory state and susceptibility to oxidative stress in humans (Yachie et al., 1999). HO-1 deficient mice acquire progressive chronic inflammatory disease (Poss and Tonegawa, 1997a) and express enhanced toxemia caused by lipopolysaccharide administration (Poss and Tonegawa, 1997b). Although the protective characteristics of HO-1 have been studied using various inflammatory models (Lee and Chau, 2002; Chora et al., 2007; Pamplona et al., 2007), the molecular mechanisms, timing, and mode of HO-1 function during disease remains largely unknown.

The mitogen-activated protein kinase (MAPK) signal transduction pathway is one of the most important regulatory mechanisms in eukaryotic cells and a central signaling cascade that is essential for the host immune response (Krachler et al., 2011). In mammals, there are at least four subfamilies of MAPKs, including the extracellular signal-regulated kinases (ERKs), the c-Jun NH_2_-terminal kinases (JNKs), the p38 isoforms (p38s), and ERK5 (Tanoue and Nishida, 2003; Turjanski et al., 2007). These kinases are organized as parallel cascades in which the activation of each component is regulated upstream and downstream by phosphorylation events (Chang and Karin, 2001). As in mouse TG cells, a large amount of phosphorylated MAPK family protein is detected in bovine TG cells. The localization of actin, its associated proteins, and phosphorylated MAPK family proteins, suggests their involvement in TG cell migration in bovine placentomes (Lang et al., 2004).

In the present study, we investigated the dephosphorylation of MAPK family proteins during *L. monocytogenes* infection in TG cells. Our results suggest that *L. monocytogenes* LLO contributes to the inhibition of MAPK signaling pathway activation and infection-associated abortion.

## Materials and Methods

### Bacterial Strains

*Listeria monocytogenes* strains EGD, Δ*hly*, and Δ*hly*::*hly* (Hara et al., 2007) and *Escherichia coli* DH5α were used in this study. Two isogenic mutants of *L. monocytogenes*, Δ*hly* and Δ*hly*::*hly* were generated from parental strain using the method of homologous recombination and the expression level of LLO was shown in a previous study (Hara et al., 2007). *L. monocytogenes* strains, which were maintained in frozen glycerol stocks, were cultured overnight in brain heart infusion (BHI) broth (Becton Dickinson, Franklin Lakes, NJ, USA) at 37°C with shaking or on BHI broth containing 1.5% agar (Wako, Osaka, Japan) at 37°C under aerobic condition. *E. coli* DH5α was cultured in Luria–Bertani (LB) broth (MO BIO Laboratories, Inc., Carlsbad, CA, USA) or on LB broth containing 1.5% agar.

### Cell Culture

A mouse TS cell line was gifted by Dr. Tanaka (Tanaka et al., 1998). Cell culture was done by using the same method described previously (Hashino et al., 2012). TS cells were cultured in a mixed medium (TS medium: mouse embryonic fibroblast-conditioned medium = 3 : 7) supplemented with 25 ng/ml fibroblast growth factor 4 (TOYOBO, Osaka, Japan) and 1 μg/ml heparin (Sigma, St. Louis, MO, USA), as described in our previous study (Hashino et al., 2012). TS medium was prepared by adding 20% (v/v) fetal bovine serum (FBS), 1 mM sodium pyruvate, 100 μM β-mercaptoethanol, and 2 mM L-glutamine to RPMI 1640 medium. TS cells were cultured in TS medium alone for 3 days at 37°C in an atmosphere containing 5% CO_2_ for inducing cell differentiation of TS cells to TG cells. TG cells were seeded in 48-well (1–2 × 10^5^ per well) or 12-well (4–8 × 10^5^ per well) tissue culture plates. After differentiation into TG cells, the cells were incubated in RPMI 1640 medium for 24 h before use.

### Immunoblotting

Immunoblotting was done by using the modified method described previously (Hashino et al., 2012). TG cells were washed twice with phosphate-buffered saline (PBS) and lysed in lysis buffer ice-cold PBS containing 1% (v/v) Triton X-100, 1 mM sodium orthovanadate (SOV), 2 mM phenylmethylsulfonyl fluoride, 100 mM sodium fluoride, and 1× Halt Protease Inhibitor Cocktail Kit (Thermo Fisher Science, Rockford, IL, USA) at 4°C for 30 min, and sonicated three times for 10 s each. the supernatants of cell lysates were collected by centrifugation (16,000 × *g*, 4°C, 20 min; Hashino et al., 2012). Protein concentrations were measured using by the Bio-Rad Protein Assay (Bio-Rad, Richmond, CA, USA). After separating 300 ng of each protein by SDS-PAGE using 12% polyacrylamide gels, the proteins were electrically transferred onto polyvinylidene difluoride (PVDF) membranes (Millipore, Billerica, MA, USA). After membranes were blocked for 2 h with 5% (w/v) non-fat dry milk in Tris-buffered saline (TBS) at room temperature, membranes were incubated with anti-mouse Phospho-c-Jun rabbit monoclonal antibody (1:1,000; Cell Signaling Technology, Danvers, MA, USA), anti-mouse c-Jun rabbit monoclonal antibody (1:1,000; Cell Signaling Technology), anti-mouse Phospho-p38 MAPK rabbit monoclonal antibody (1:1,000; Cell Signaling Technology), anti-mouse p38 MAPK rabbit monoclonal antibody (1:1,000; Cell Signaling Technology), anti-mouse Phospho-p44/42 MAPK rabbit monoclonal antibody (1:1,000; Cell Signaling Technology), anti-mouse p44/42 MAPK rabbit monoclonal antibody (1:1,000; Cell Signaling Technology), anti-mouse HO-1 rabbit polyclonal antibody (1:2,000; Stressgen, BC, Canada), anti-mouse TLR2 rat monoclonal antibody (1:200; R&D Systems Inc., Minneapolis, MN, USA), or anti-mouse β-actin antibody (1:5,000; Sigma) at 4°C overnight, as appropriate. After washing with TBS containing 0.02% (v/v) Tween 20, membranes were incubated with horseradish peroxidase-conjugated secondary antibody (0.01 μg/ml) at room temperature for 1 h and immunoreactions were visualized using the enhanced chemiluminescence detection system (GE Healthcare Life Science, Little Chalfont, UK). Multi gauge software (Fujifilm Life Science, Tokyo, Japan) was used for the quantification of the bands and all protein levels were normalized to the β-actin levels after densitometric scanning of the membranes by LAS-4000 mini Imaging System (Fujifilm Life Science).

### Efficiency of Bacterial Internalization within Cultured Cells

Bacterial internalization assay was performed by using the modified method described previously (Hashino et al., 2012). TG cells were infected with each bacterial strains at a multiplicity of infection (MOI) of either 10 or 5 by centrifugation at 150 × *g* for 10 min at room temperature. To measure bacterial internalization efficiency after 30 min of incubation at 37°C in atmosphere containing 5% CO_2_, the cells were washed once with RPMI 1640 medium and then replaced with new media containing gentamicin (50 μg/ml) to kill any remaining extracellular bacteria. After 30 min of incubation at 37°C in atmosphere containing 5% CO2, The cells were then washed three times with PBS and lysed with cold distilled water. The serial dilution of cell lysates was spread on BHI or LB plates for measuring colony-forming unit (CFU) values (Hashino et al., 2012). Dimethyl sulfoxide (DMSO, Wako), SP600125 (Wako), SB203580 (Wako), or U0126 (Wako) were added 1 h before infection. SOV (Sigma) was added immediately before *L. monocytogenes* infection. These inhibitors were dissolved in 1% DMSO (v/v). Trypan blue dye exclusion staining was used for cell viability analysis.

### Detection of Cytotoxicity

Cytotoxicity was detected using the Cytotoxicity Detection KitPLUS (LDH) according to the manufacturer’s instructions (Roche Applied Science, Upper Bavaria, Germany). *L. monocytogenes* was deposited onto TG cells at a MOI of 10 or 5 by centrifugation at 150 × *g* for 10 min at room temperature. To measure cytotoxicity, the infected cells were incubated at 37°C in atmosphere containing 5% CO_2_ for 30 min, after which the cells were incubated with RPMI 1640 medium containing gentamicin (50 μg/ml) for 30 min to remove extracellular bacteria. The cells were washed once with RPMI 1640 medium, and then incubated in RPMI 1640 medium at 37°C in atmosphere containing 5% CO_2_ for 6 h. SOV (100 μM) was added immediately before *L. monocytogenes* infection.

### Determination of Cell Death

Cell death was assessed using the JC-1 Mitochondrial Membrane Potential Assay Kit (Cayman Chemical, Ann Arbor, MI, USA) according to the manufacturer’s instructions. Bacteria were deposited onto TG cells on coverslips by centrifugation (150 × *g*, 10 min, room temperature), incubated at 37°C in atmosphere containing 5% CO_2_ for 30 min, and then incubated in RPMI 1640 medium containing gentamicin (50 μg/ml) for 30 min. The cells were washed once with RPMI 1640 medium, and then incubated in RPMI 1640 medium at 37°C in atmosphere containing 5% CO_2_ for 6 h. For JC-1 staining, cells were incubated with JC-1 staining solution at 37°C for 15 min. The samples were washed twice with assay buffer. Fluorescent images were obtained using a FluoView FV100 confocal laser scanning microscope (Olympus, Tokyo, Japan). SOV (100 μM) was added immediately before *L. monocytogenes* infection. Mitochondrial membrane potential, ΔΨ_m_, is used as an indicator of cell health. In this system, healthy cells show red fluorescence, while apoptotic or unhealthy cells show green fluorescence (Tachibana et al., 2008, 2011).

### Small Interfering RNA (siRNA)

Small interfering RNA (siRNA) duplexes used for silencing mouse MRC1 (target sequence: 5′-CAGCATGTGTTTCAAACTGTA-3′), TLR2 (target sequence: 5′-TTGGATGTTAGTAACAACAAT-3′), and AllStars Negative Control siRNA were purchased from Qiagen (Hilden, Germany). TG cells were transiently transfected with MRC1 siRNA using Lipofectamine RNAiMAX (Invitrogen, Carlsbad, CA, USA) with or without siRNAs at a final concentration of 36 nM and transfected with TLR2 siRNA using X-tremeGENE siRNA Transfection Reagent (Roche Applied Science) with or without siRNAs at a final concentration of 10 nM (Hashino et al., 2012).

### RNA Isolation and qPCR Analysis of MRC1

RNA isolation was done by using the same method described previously (Tachibana et al., 2011). Total RNA for real-time PCR was prepared from TG cells by using the RNA Purification Kit (Qiagen), and the purified RNA samples were kept at –30°C until use. RNA was quantitated by absorption at 260 nm using a SmartSpec 3000 spectrophotometer (Bio-Rad). One microgram of total RNA was used to synthesize cDNA using a ReverTra Ace quantitative PCR (qPCR) reverse transcriptase (RT) kit (TOYOBO). Real-time PCR was performed using Thunderbird SYBR qPCR Mix (TOYOBO). The following primer sets were purchased from TAKARA BIO (Shiga, Japan): MRC1, forward 5′-AGCTTCATCTTCGGGCCTTTG-3′ and reverse 5′-GGTGACCACTCCTGCTGCTTTAG-3′; and β-actin, forward 5′-TGACAGGATGCAGAAGGAGA-3′ and reverse 5′-GCTGGAAGGTGGACAGTGAG-3′. All data were normalized to β-actin.

### Murine Experiments

Eight to 12 week-old female ICR mice were mated individually with male ICR mice. The parent mice were obtained from Kyudo Co., Ltd. (Saga, Japan). All mice were maintained under specific pathogen-free conditions in sterile cages housed in a ventilated isolator. Fluorescent lights were cycled 12 h on/12 h off, and ambient temperature (23 ± 1°C) and relative humidity (40–60%) were regulated. A vaginal plug was observed at day 0.5 of gestation. The normal gestational time for these mice is 19 days.

### Virulence in Pregnant Mice

Mouse virulence assay was done by using the modified method described previously (Tachibana et al., 2011). Groups of three pregnant mice were infected intravenously at 7.5 days of gestation with approximately 2 × 10^4^ cells of *L. monocytogenes* strains in 0.1 ml PBS. To check the progression of the disease, infected mice were monitored by a veterinarian every 3–4 h during the day phase (7:00 am to 7:00 pm). At 18.5 days of gestation, their livers and spleens were removed, weighed and homogenized in PBS. The tissue homogenates were serially diluted with PBS and plated on BHI agar plates to count the number of CFUs. Fetuses were classified as alive if there was a heartbeat and as dead if there was no heartbeat (Tachibana et al., 2011).

### Statistical Analyses

Statistical analyses were performed using the ANOVA test with *post hoc* Tukey–Kramer test or Student’s *t*-test. Statistically significant differences between groups are indicated by asterisks (^∗^*P* < 0.05). Values represent the means from three assays and the error bars represent standard deviations (SD).

## Results

### Dephosphorylation of MAPK Family Proteins by *L. monocytogenes* Infection of TG Cells

**FIGURE 1:**
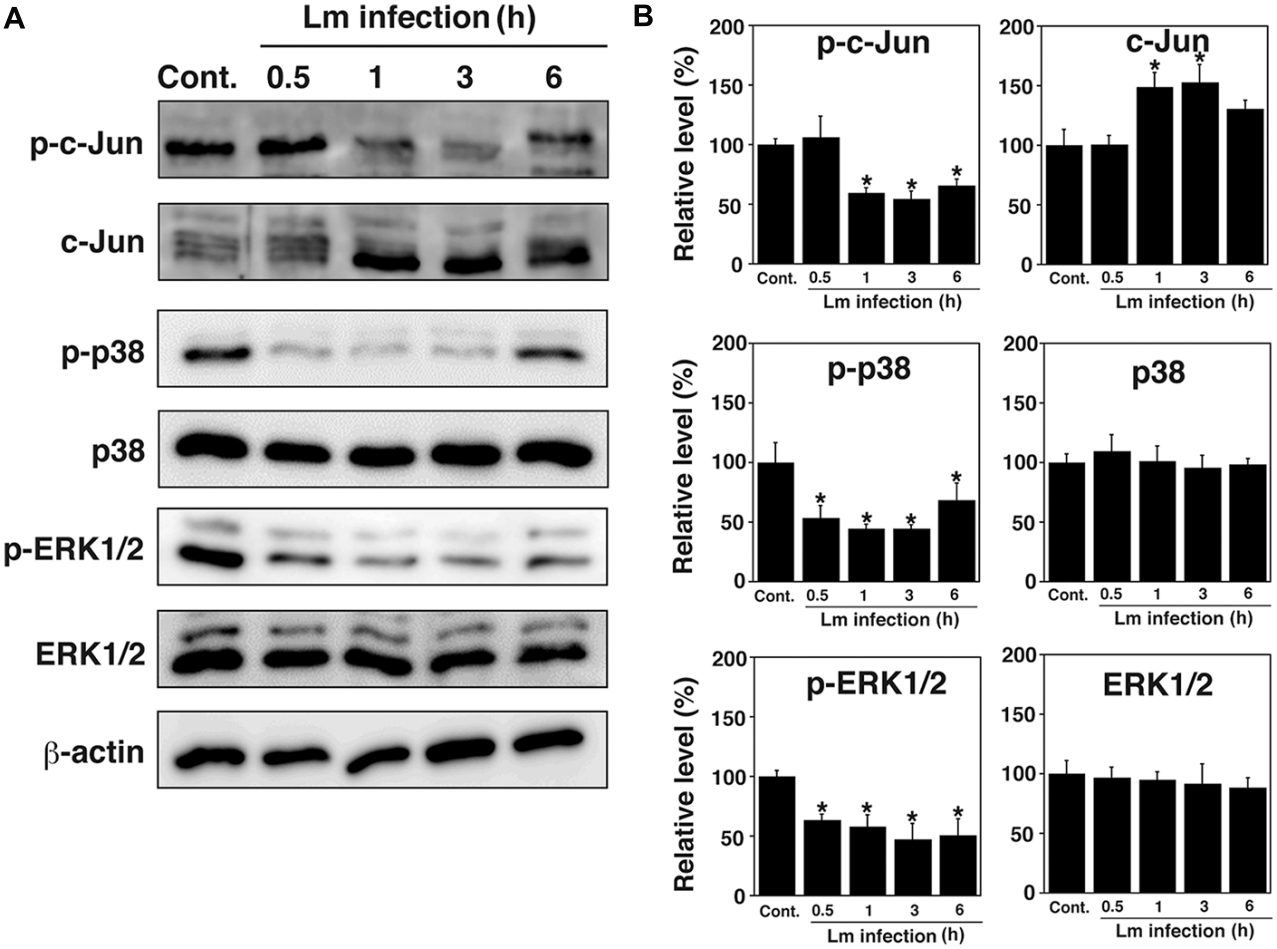
**Dephosphorylation of MAPK family proteins in trophoblast giant (TG) cells by *Listeria monocytogenes* infection. (A)** Expression of MAPK family proteins in TG cells infected with *L. monocytogenes*. TG cells were infected with or without (Cont.) *L. monocytogenes* for the indicated number of hours. Production of the indicated proteins was detected by immunoblotting. β-actin was used as an immunoblotting control. Phosphorylated c-Jun, p38, and ERK1/2 are designated p-c-Jun, p-p38, and p-ERK1/2, respectively. **(B)** Relative intensity levels of p-c-Jun, c-Jun, p-p38, p-38, p-ERK, and ERK. The intensity levels of all MAPK family proteins were measured using Multi gauge software and normalized by the value of β-actin. Relative values to the intensity without infection (Cont.) were shown. All values represent the means and SD of three assays. ^∗^*P* < 0.05 compared with Cont. by *post hoc* Tukey–Kramer test.

Because the MAPK pathway is involved in the infection of host cells by bacterial pathogens (Krachler et al., 2011), the phosphorylation of c-Jun, p38, and ERK1/2 in TG cells after *L. monocytogenes* infection was analyzed by immunoblotting. Phosphorylation of c-Jun, p38, and ERK1/2 was observed in uninfected control TG cells, whereas dephosphorylation of these proteins was observed in infected TG cells (**Figures 1A,B**). These proteins were dephosphorylated at different times during bacterial infection. Dephosphorylation of c-Jun, p38, and ERK1/2 was observed 1–6 h, 0.5–6 h, and 0.5–6 h after infection, respectively (**Figures 1A,B**).

### Dephosphorylation of MAPK Family Proteins Induces Downregulation of HO-1

**FIGURE 2:**
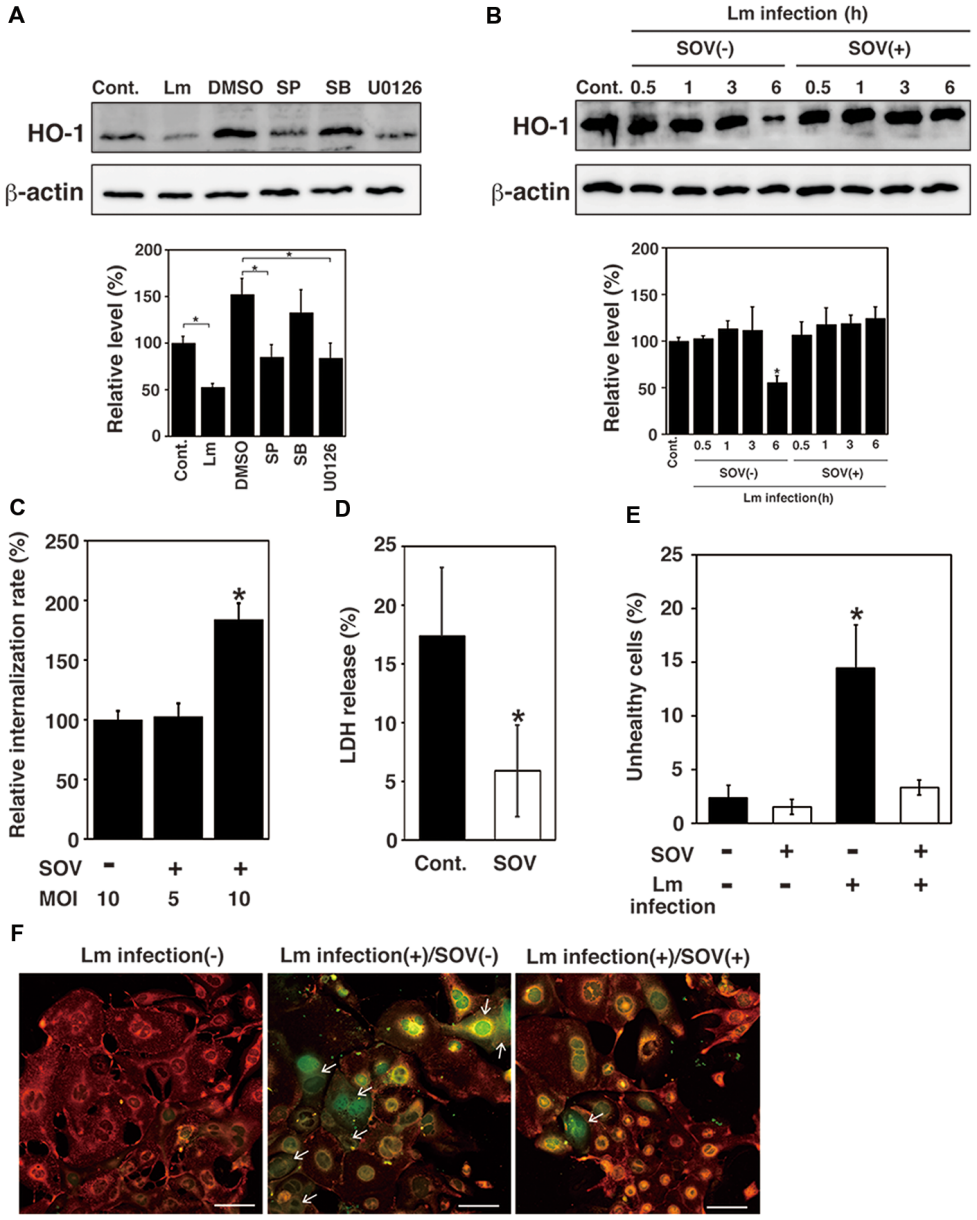
**Dephosphorylation of MAPK family proteins induces downregulation of HO-1 and TG cell death. (A)** Effect of MAPK inhibitor treatment on HO-1 expression. TG cells were infected with *L. monocytogenes* (Lm) for 6 h, treated with or without (Cont.) SP600125 (SP; JNK inhibitor, 25 μM), SB203580 (SB; p38 inhibitor, 25 μM), U0126 (ERK1/2 inhibitor, 25 μM), or DMSO 1 h before infection, respectively. Expression of HO-1 was detected by immunoblotting. β -actin was used as an immunoblotting control. **(B)** Effect of SOV treatment on HO-1 expression. TG cells were infected with or without (Cont.) *L. monocytogenes* in the presence (SOV+) or absence (SOV–) of SOV (100 μM) for the indicated number of hours. HO-1 expression was detected by immunoblotting. β-actin was used as an immunoblotting control. **(A,B)** The intensity levels of HO-1 expression were measured using Multi gauge software and normalized by the value of β-actin. Relative values to the intensity without infection (Cont.) were shown. All values represent the means and SD of three assays. ^∗^*P* < 0.05 compared with Cont. by *post hoc* Tukey–Kramer test. **(C)** Relative bacterial internalization rate. TG cells were infected with *L. monocytogenes* in the presence (SOV+) or absence (SOV–) of SOV (100 μM), for 30 min at an multiplicity of infection (MOI) of 10 or 5. All values represent the means and SD of triplicate samples from three assays. ^∗^*P* < 0.05 compared with bacterial internalization in TG cells lacking SOV by *post hoc* Tukey–Kramer test. **(D)** Detection of cytotoxicity by LDH release. TG cells were infected with *L. monocytogenes* in the presence (SOV) or absence (Cont.) of SOV (100 μM) at an MOI of 10 (SOV–) or 5 (SOV+). The amount of LDH released was measured after 6 h of infection. All values represent the means and SD of triplicate samples from three assays. ^∗^*P* < 0.05 compared with Cont. by Student’s *t*-test. **(E)** Detection of cell death. TG cells were infected with *L. monocytogenes* in the presence (SOV+) or absence (SOV–) of SOV (100 μM) for 30 min at an MOI of 10 (SOV–) or 5 (SOV+). Cell death was determined using the JC-1 Mitochondrial Membrane Potential Assay Kit after 6 h of infection. The total number of live or dead cells was determined by examination of 100 TG cells per coverslip. All values represent the means and SD of three assays. ^∗^*P* < 0.05 compared with control by *post hoc* Tukey–Kramer test. **(F)** Fluorescence microscopic images of cell death. Red or green fluorescence indicates healthy and unhealthy cells, respectively. Arrows indicate dead cells. Bar represents 100 μm.

**FIGURE 3:**
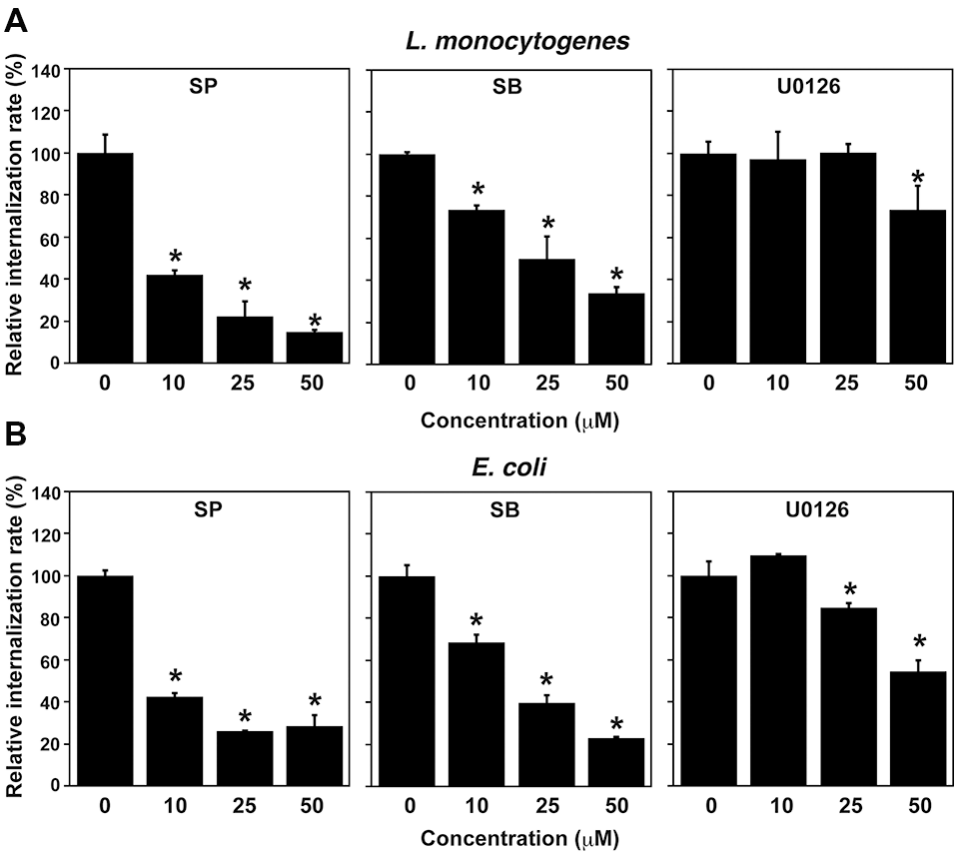
**Treatment with MAPK inhibitors inhibits *L. monocytogenes* infection in TG cells. (A)** Effect of MAPK inhibitor treatment on *L. monocytogenes* infection. TG cells were treated with SP600125 (SP; JNK inhibitor), SB203580 (SB; p38 inhibitor), or U0126 (ERK1/2 inhibitor) at the indicated concentration for 1 h and then infected with *L. monocytogenes* for 30 min at an MOI of 10. **(B)** Effect of MAPK inhibitor treatment on uptake of *Escherichia coli*. TG cells were treated with MAPK inhibitors as described above and then infected with *E. coli* for 30 min at an MOI of 10. **(A,B)** All values represent the means and SD of triplicate samples from three identical experiments. ^∗^*P* < 0.05 compared with bacterial internalization in TG cells without treatment by *post hoc* Tukey–Kramer test.

*Listeria monocytogenes* internalize into host cells and induce host cell death (Disson and Lecuit, 2013). In a previous study, we suggested that HO-1 inhibits cell death caused by *L. monocytogenes* infection (Tachibana et al., 2011). To investigate whether MAPK contributes to expression of HO-1 in TG cells, the effect of MAPK inhibitors on HO-1 expression in TG cells was analyzed by immunoblotting. HO-1 expression was observed in uninfected TG cells, whereas the HO-1 expression was decreased in infected TG cells (**Figure 2A**). HO-1 expression was decreased by the treatment of uninfected TG cells with SP600125 (JNK inhibitor) and U0126 (ERK1/2 inhibitor), but not SB203580 (p38 inhibitor; **Figure 2A**). DMSO may affect HO-1 expression slightly. It was difficult to analyze HO-1 expression after bacterial infection under the MAPK inhibitor treatment, because treatment of MAPK inhibitor inhibited bacterial internalization (see **Figure 3**). These results suggest that the MAPK pathway contributes to HO-1 expression in TG cells.

We next investigated that the effect of SOV, an inhibitor of protein tyrosine phosphatases, on HO-1 expression in infected TG cells by immunoblotting. A decrease in HO-1 expression was observed in infected but untreated TG cells. The downregulation of HO-1 expression caused by bacterial infection was inhibited by SOV treatment (**Figure 2B**). We also observed that dephosphorylation of MAPK family proteins was inhibited by SOV treatment (data not shown). In addition, we assessed the effect of SOV treatment on bacterial internalization into TG cells. The results showed that bacterial internalization into TG cells was significantly increased by SOV treatment (**Figure 2C**). SOV treatment did not affect cell viability that was confirmed by trypan blue dye exclusion staining.

To examine whether protein dephosphorylation induced by bacterial infection contributes to cell death, infection of *L. monocytogenes* to TG cells was done with or without SOV treatment and the rate of cytotoxicity and cell death was analyzed by measuring LDH release and the mitochondrial membrane potential. However, in this experimental system, cells with low mitochondrial membrane potential were detected as unhealthy cells (**Figure 2F**). Since SOV treatment increased bacterial internalization in TG cells (**Figure 2C**), we reduced the MOI to 5 from 10 when TG cells were treated with SOV to equalize the number of internalized bacteria. Therefore, MOI in SOV untreated and treated cells in **Figures 2B,D,E** were 10 and 5, respectively. In this condition, the number of internalized bacterial was same as shown in **Figure 2C**. Treatment with SOV inhibited cell death induced by *L. monocytogenes* infection in TG cells, which was not observed in infected, untreated TG cells (**Figures 2D,E**).

### Inhibition of MAPK Family Proteins Impairs Bacterial Internalization into TG Cells

Because HO-1 expression was decreased by treatment with JNK and ERK1/2 inhibitors, we next examined the effect of treatment with these inhibitors on bacterial internalization into TG cells. Internalization of *L. monocytogenes* into TG cells was decreased by treatment with SP600125 (JNK inhibitor), SB203580 (p38 inhibitor), and U0126 (ERK1/2 inhibitor) in a dose-dependent manner (**Figure 3A**). Moreover, uptake of *E. coli* by TG cells was inhibited by treatment with these inhibitors (**Figure 3B**). These results suggest that the MAPK pathway contributes to the bacterial internalization into TG cells. Inhibitor treatments did not affect cell viability that was confirmed by trypan blue dye exclusion staining.

### Expression of MAPK Family Proteins in TG Cells upon MRC1 and TLR2 Knockdown

**FIGURE 4:**
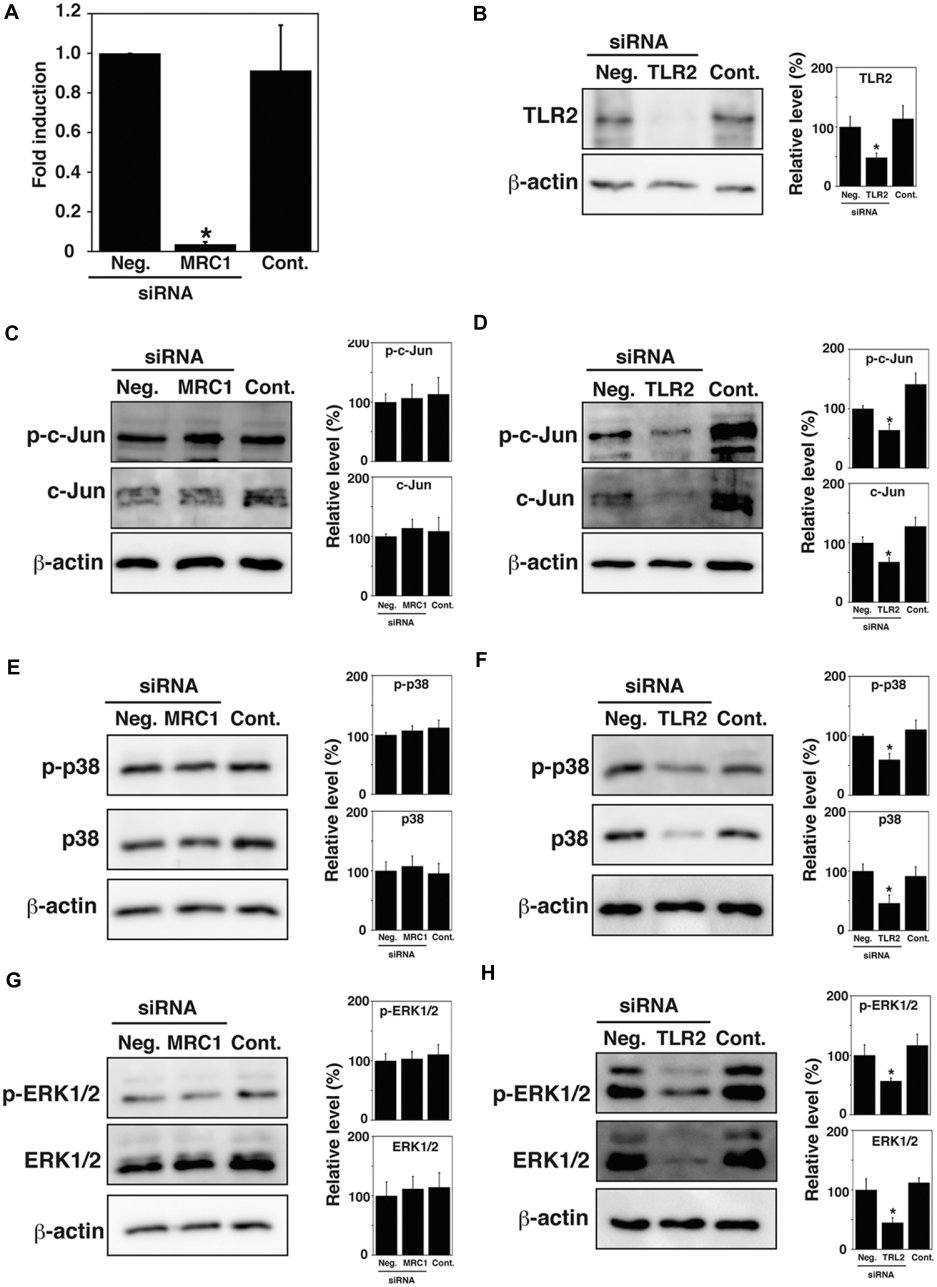
**Expression of MAPK family proteins in MRC1 and TLR2 knock-down TG cells. (A,B)** Expression of MRC1 and TLR2. TG cells were treated for 48 h with or without (Cont.) siRNA-targeting MRC1 **(A)** or TLR2 **(B)**. AllStars Negative Control siRNA (Neg.) was used for a negative control. Production of the indicated proteins was monitored by qPCR **(A)** or immunoblotting **(B)**. β-actin was used as a control. **(C,D)** Detection of c-Jun and phosphorylated c-Jun (p-c-Jun) in MRC1 **(C)** or TLR2 **(D)** knock-down TG cells by immunoblotting. **(E,F)** Detection of p38 and phosphorylated p38 (p-p38) in MRC1 **(E)** or TLR2 **(F)** knock-down TG cells by immunoblotting. **(G,H)** Detection of ERK1/2 and phosphorylated ERK1/2 (p-ERK1/2) in MRC1 **(G)** or TLR2 **(H)** knock-downed TG cells by immunoblotting. **(B–H)** The intensity levels of each protein expression were measured using Multi gauge software and normalized by the value of β-actin. Relative values to the intensity of Neg. were shown. **(A–H)** All values represent the means and SD of three assays. ^∗^*P* < 0.05 compared with Neg. by *post hoc* Tukey–Kramer test.

Previous studies have reported that Mannose receptor, C type 1 (MRC1) and Toll-like receptor 2 (TLR2) mediate bacterial uptake by TG cells (Watanabe et al., 2010; Hashino et al., 2012). We hypothesized that activation or expression of MAPK family proteins may be mediated by MRC1 and TLR2. To examine whether MRC1 and TLR2 contribute to the activation or expression of MAPK family proteins, the phosphorylation and expression of c-Jun, p38, and ERK1/2 in TG cells were analyzed by immunoblotting after the knockdown of these receptors. The amounts of endogenous MRC1 and TLR2 were reduced by transfecting TG cells with MRC1- or TLR2-specific siRNA duplexes. MRC1 expression was significantly reduced and TLR2 expression was no longer detectable 48 h after transfection of TG cells with MRC1- or TLR2-specific siRNA, however, transfection with control siRNA had no effect on expression (**Figures 4A,B**). Because MRC1 is difficult to detect by immunoblotting, MRC1 expression was analyzed by reverse transcription quantitative PCR. Although MRC1 knockdown did not affect the phosphorylation or expression of c-Jun, p38, or ERK1/2 in TG cells (**Figures 4C,E,G**), TLR2 knockdown reduced the expression of c-Jun, p38, and ERK1/2 (**Figures 4D,F,H**). These results suggest that TLR2 plays critical roles in signal transduction through the MAPK pathway in TG cells.

### Infection-associated Abortion and Dephosphorylation of MAPK Family Proteins Depends on LLO

**FIGURE 5:**
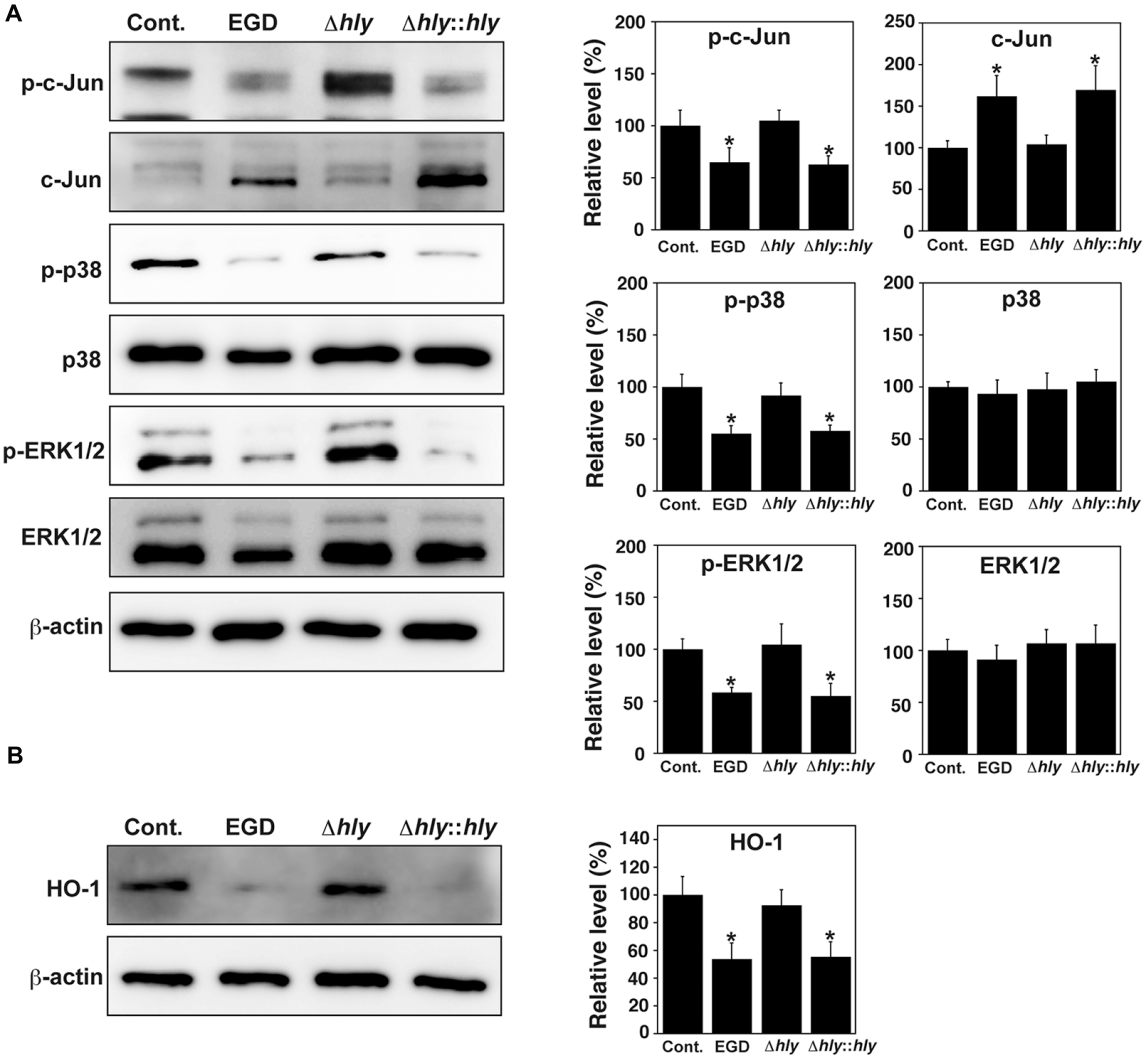
**LLO contributes to dephosphorylation of MAPK family proteins and downregulation of HO-1. (A)** Expression of MAPK family proteins. TG cells were infected with or without (Cont.) *L. monocytogenes* wild-type strain EGD, LLO deletion mutant (Δ*hly*), or LLO-complemented strain (Δ*hly*::*hly*) for 3 h. Expression of the indicated proteins was detected by immunoblotting. β-actin was used as an immunoblotting control. Phosphorylated c-Jun, p38, and ERK1/2 are indicated as p-c-Jun, p-p38, and p-ERK1/2, respectively. **(B)** Expression of HO-1. TG cells were infected with the *L. monocytogenes* strains described above for 6 h. Expression of HO-1 was detected by immunoblotting. β-actin was used as an immunoblotting control. **(A,B)** The intensity levels of each protein expression were measured using Multi gauge software and normalized by the value of β-actin. Relative values to the intensity without infection (Cont.) were shown. All values represent the means and SD of three assays. ^∗^*P* < 0.05 compared with Cont. by *post hoc* Tukey–Kramer test.

The thiol-activated, cholesterol-dependent, pore-forming toxin LLO is a major virulence factor of *L. monocytogenes* (Hernández-Flores and Vivanco-Cid, 2015). To investigate whether LLO contributes to the dephosphorylation of MAPK family proteins, TG cells were infected with wild-type *L. monocytogenes* EGD, an LLO deletion mutant (Δ*hly*), and an LLO-complemented strain (Δ*hly*::*hly*). Subsequently, the phosphorylation of c-Jun, p38, and ERK1/2 in these cells was analyzed by immunoblotting. Phosphorylation of c-Jun, p38, and ERK1/2 was observed in uninfected TG cells and in TG cells infected with LLO deletion mutant. Dephosphorylation of these proteins was observed in TG cells infected with wild-type EGD and with LLO-complemented strain (**Figure 5A**).

To investigate whether LLO contributes to the expression of HO-1 in TG cells, TG cells were infected with the three strains described above, and HO-1 expression in these cells was analyzed by immunoblotting. HO-1 expression was observed in uninfected TG cells and in TG cells infected with the LLO deletion mutant. HO-1 expression was decreased in TG cells infected with wild-type EGD and with the LLO-complemented strain infection (**Figure 5B**).

**FIGURE 6:**
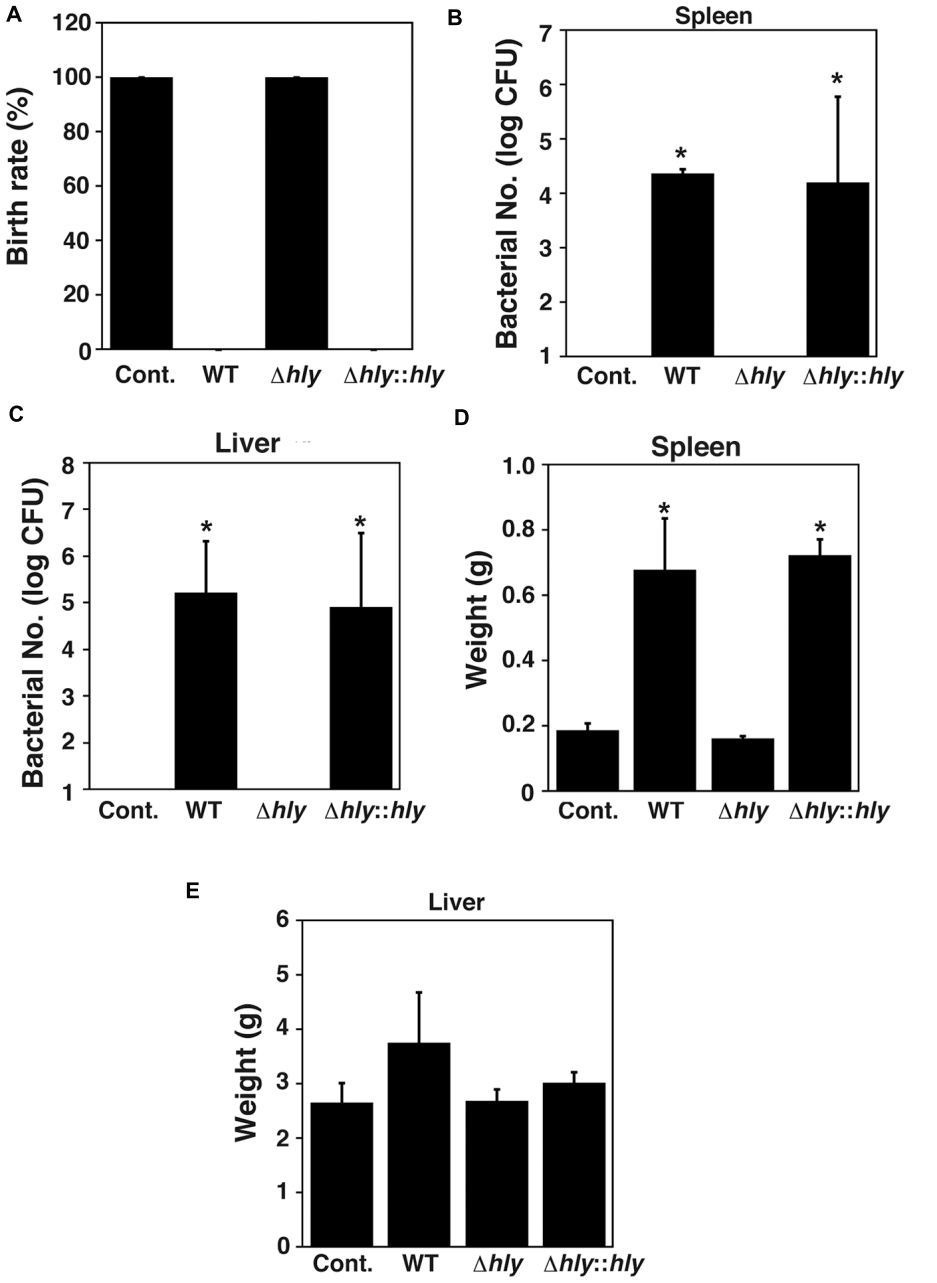
**LLO contributes to infection-associated abortion. (A)** Birth rate. Groups of three pregnant animals were infected intravenously at 7.5 days of gestation with approximately 2 × 10^4^ cells of *L. monocytogenes* wild-type strain EGD, LLO deletion mutant (Δ*hly*), and LLO-complemented strain (Δ*hly*::*hly*). **(B,C)** Number of bacteria in the spleen **(B)** and liver **(C)** of mice infected with the *L. monocytogenes* strains described above. At 18.5 days of gestation, their spleens and livers were removed and homogenized. The serially diluted tissue homogenates were plated on BHI agar plates and the number of CFU was determined. **(D,E)** Weights of spleen **(D)** and liver **(E)** from mice infected with the *L. monocytogenes* strains described above. **(B–E)** All values represent the means and SD (*n* = 9). ^∗^*P* < 0.05 compared with Cont. by *post hoc* Tukey–Kramer test.

Further, we investigated the role of LLO in infection-associated abortion induced by *L. monocytogenes*. In order to construct a mouse model of infection-associated abortion induced by *L. monocytogenes*, the numbers of fetuses aborted by infected mice were counted on day 18.5 of gestation. All fetuses were aborted when the mice were infected with wild-type *L. monocytogenes* on day 7.5 of gestation. Almost fetuses were still alive when the mice were infected on days 10.5 and 11.5 of gestation. No abortion was observed when the mice were infected on day 14.5 of gestation. Therefore, we analyzed abortion caused by *L. monocytogenes* infection on day 7.5 of gestation. Infection-associated abortion was observed in pregnant mice infected with wild-type EGD and the LLO-complemented strain, whereas the LLO deletion mutant did not induce infection-associated abortion (**Figure 6A**). We next examined bacterial colonization of the spleen and liver. Replicating bacteria were observed in both the spleen and liver of mice infected with wild-type EGD and the LLO-complemented strain, whereas replicating bacteria were undetectable in the spleen and liver of mice infected with the LLO deletion mutant (**Figures 6B,C**). Infection with wild-type EGD and the LLO-complemented strain significantly increased the weight of the spleen (**Figure 6D**), while bacterial infection had no significant effect on the weight of the liver (**Figure 6E**).

## Discussion

MAPK activation induces the expression of multiple genes that regulate inflammatory responses. Intracellular pathogens manipulate MAPK pathways to increase their virulence (Krachler et al., 2011; Arthur and Ley, 2013). Several bacterial pathogens, including *Mycobacterium tuberculosis*, activate MAPK pathways and promote invasion of host cells (Schorey and Cooper, 2003). In contrast, *Yersinia* sp. and *Shigella* sp. inhibit phosphorylation of MAPK and negatively regulate proinflammatory responses (Orth et al., 1999; Reiterer et al., 2011). In this study, dephosphorylation of c-Jun, p38, and ERK1/2 was observed during *L. monocytogenes* infection of TG cells. When *L. monocytogenes* is internalized into endothelial cells, p38 is activated and IL-8 secretion is induced in a nucleotide-binding oligomerization domain (Nod) 1-dependent manner (Opitz et al., 2006). Although MAPK plays an important role in immune responses to *L. monocytogenes* infection, its role in the internalization of bacteria into TG cells was unclear. YopJ, a *Yersinia* sp. virulence factor, is injected directly into host cells through a needle-like complex called the type III secretion system (Espinosa and Alfano, 2004). YopJ inhibits MAPK pathways and the NF-κB pathway by preventing the activation of MAP kinase kinase (MKK) and IκB kinase b (IKKb) and promotes host cell death (Orth et al., 1999). Since *L. monocytogenes* infection also induces cell death in TG cells (Tachibana et al., 2011), dephosphorylation of c-Jun, p38, and ERK1/2 is thought to contribute to TG cell death induced by bacterial infection.

MAPK pathways are also known to be associated with the production of HO-1 (Paine et al., 2010). HO-1 plays key roles in cytoprotection, antioxidation, and anti-inflammation (Hegazi et al., 2005; Nakahira et al., 2006). During spontaneous abortion, HO-1 is downregulated at the fetal-maternal interface in both human and mice (Yachie et al., 1999). We reported previously that HO-1 contributed to abortion caused by *L. monocytogenes* and *B. abortus* infection (Tachibana et al., 2008, 2011). *B. abortus* is a Gram-negative, intracellular, zoonotic bacterium. These bacteria cause downregulation of HO-1 in the placenta, resulting in abortion (Tachibana et al., 2008). In this study, we showed that inhibition of c-Jun and ERK1/2 pathway causes downregulation of HO-1. Inhibition of protein dephosphorylation by SOV treatment blocks the downregulation of HO-1 and TG cell death caused by bacterial infection. Furthermore, our results suggested that TG cells survived if downregulation of HO-1 was not induced, although a large number of bacteria were internalized into TG cells. Besides, not only down regulation of HO-1 but also bacterial infection is necessary for induction of TG cell death (Tachibana et al., 2008, 2011). Therefore, it is speculated that the death of TG cells resulting from *L. monocytogenes* infection may be caused by the downregulation of HO-1 due to the dephosphorylation of MAPK family proteins. Therefore, dephosphorylation of MAPK family proteins by *L. monocytogenes* infection may be first step to infection-associated abortion, but detail mechanism of infection-associated abortion is still unclear.

Since SOV treatment enhanced internalization of *L. mono cytogenes* into TG cells, phosphorylated proteins contributed to bacterial internalization. Phosphatidylinositol 3-kinase (PI3K)/Akt pathway is activated by binding to phosphorylated proteins (Wang et al., 2015), and our previous study showed that the activation of PI3K/Akt pathway contribute to *L. monocytogenes* infection in TG cells (Tachibana et al., 2015). In this study, we demonstrated that phosphorylation of MAPK family proteins also contributes to bacterial internalization. This data indicates that internalization of bacteria is controlled through various intracellular signaling pathway including MAPK and PI3K/Akt pathway. Although activation of the MAPK pathway is required to enhance bacterial internalization, *L. monocytogenes* induced dephosphorylation of MAPK family proteins in TG cells. The level of MAPK activity in TG cells is usually high, allowing TG cells to internalize macromolecules, such as bacteria. Therefore, the ability of TG cells to internalize additional bacteria would decrease after *L. monocytogenes* infection. This phenomenon is believed to prevent rapid cell death by sequential bacterial infection. This method of infection control by *L. monocytogenes* in TG cells may be important for intracellular bacterial growth and the induction of infection-associated abortion.

In our previous studies, we showed that several receptors located on TG cells contribute to *L. monocytogenes* infection, such as MRC1, TLR2, and class B scavenger receptor type 1 (SR-B1; Watanabe et al., 2010; Hashino et al., 2012). Knockdown of these receptors inhibited the uptake of *L. monocytogenes* by TG cells. Although it is well known that signal transduction mediated by TLR2 activates the MAPK pathway (Re and Strominger, 2001), the role of MRC1 during the signal transduction remains unclear. The results of this study showed that TLR2 contributes to the expression of MAPK family proteins in TG cells, but that MRC1 was not involved. Since MAPK signaling pathway seemed to play important role in internalization of bacteria and cell death, TLR2 may be a key factor in controlling *L. monocytogenes* infection in TG cells.

Listeriolysin O is known as a major virulence factor and contributes to bacterial escape from the phagocytic vacuole in the host cell. Recent studies have revisited the role of LLO and are providing new functions of LLO (Hamon et al., 2012). Infection with *L. monocytogenes* causes fragmentation of the host mitochondrial network through the pore-forming action of its toxin LLO before bacterial entry (Stavru et al., 2011b). LLO has been described as highly lytic when applied to nucleated cells and can induce a wide range of cell death types. *L. monocytogenes* infection induces apoptosis in the cells of the spleen, lymph nodes, liver, and brain (Hernández-Flores and Vivanco-Cid, 2015). The *in vitro* results of LLO-mediated induction of apoptosis are in agreement with *in vivo* observations (Carrero et al., 2004). During *L. monocytogenes* infection of a macrophage cell line, the bacterium induces expansion of the endoplasmic reticulum (ER) and initiates a stress response to unfolded proteins (unfolded protein response or UPR). The induction of this ER stress response is dependent on the production of LLO. ER stress limits the number of intracellular bacterial, but sustained ER stress results in apoptotic cell death (Pillich et al., 2012). In this study, we showed that dephosphorylation of MAPK family proteins during *L. monocytogenes* infection in TG cells is dependent on LLO. A mutant lacking LLO did not induce infection-associated abortion, suggesting that the modulation of infection in the placenta should be advantageous toward *L. monocytogenes* infection.

## Conclusion

Dephosphorylation of MAPK family proteins by *L. monocyto genes* in TG cells contributes to TG cell death induced by bacterial infection. Thus, LLO plays an important role in both TG cell death and infection-associated abortion.

## Author Contributions

Conceived and designed experiments: MH, MW.

Performed the experiments: MH, MT, TN, KW, TS.

Analyzed the data: MH, MT, TN, HH, KT, MM, KW, TS, MW.

Contributed reagents/materials/analysis tools: HH, KT, MM.

Wrote the paper: MH, MW.

All authors read and approved the final manuscript.

## Ethics Statement

This study was performed in strict accordance with recommendations in the Guidelines for Proper Conduct of Animal Experiments stipulated by the Science Council of Japan. All experimental protocols involving the use of animals were approved by the Animal Research Committee of Yamaguchi University (Permit Number: 141). Animal studies were performed in compliance with the Yamaguchi University Animal Care and Use guidelines. The mice were sacrificed by overdose of isoflurane and all efforts were made to minimize suffering by using isoflurane anesthesia.

## Conflict of Interest Statement

The authors declare that the research was conducted in the absence of any commercial or financial relationships that could be construed as a potential conflict of interest.
